# Microinvasion in hepatocellular carcinoma: predictive factor and application for definition of clinical target volume for radiotherapy

**DOI:** 10.1186/s12957-024-03399-1

**Published:** 2024-05-08

**Authors:** Huamei Yan, Lili Liu, Donghui Wang, Jianliang Xu, Yaling Sun, Shaobo Liang, Yongheng Huang, Xinzhao Hao, Nan Lin, Xiangying Xu

**Affiliations:** 1 Department of Radiation Oncology, The Third Affiliated Hospital of Sun Yat-sen University, Guangzhou, 510630 China; 2 State Key Laboratory of Oncology in South China, Collaborative Innovation Center for Cancer Medicine, Sun Yat-sen University Cancer Center, Guangzhou, 510060 China; 3 Department of Pathology, Sun Yat-sen University Cancer Center, Guangzhou, 510060 China; 4 Department of Hepatobiliary Surgery, The Third Affiliated Hospital of Sun Yat-sen University, Guangzhou, 510630 China

**Keywords:** Hepatocellular carcinoma, Microinvasion, Clinicopathologic correlation, Radiotherapy, Clinical target volume

## Abstract

**Background:**

To investigate the correlation between microinvasion and various features of hepatocellular carcinoma (HCC), and to clarify the microinvasion distance from visible HCC lesions to subclinical lesions, so as to provide clinical basis for the expandable boundary of clinical target volume (CTV) from gross tumor volume (GTV) in the radiotherapy of HCC.

**Methods:**

HCC patients underwent hepatectomy of liver cancer in our hospital between July 2019 and November 2021 were enrolled. Data on various features and tumor microinvasion distance were collected. The distribution characteristics of microinvasion distance were analyzed to investigate its potential correlation with various features. Tumor size compared between radiographic and pathologic samples was analyzed to clarify the application of pathologic microinvasion to identify subclinical lesions of radiographic imaging.

**Results:**

The average microinvasion distance was 0.6 mm, with 95% patients exhibiting microinvasion distance less than 3.0 mm, and the maximum microinvasion distance was 4.0 mm. A significant correlation was found between microinvasion and liver cirrhosis (*P* = 0.036), serum albumin level (*P* = 0.049). Multivariate logistic regression analysis revealed that HCC patients with cirrhosis had a significantly lower risk of microinvasion (*OR* = 0.09, 95%CI = 0.02 ~ 0.50, *P* = 0.006). Tumor size was overestimated by 1.6 mm (95%CI=-12.8 ~ 16.0 mm) on radiographic size compared to pathologic size, with a mean %Δsize of 2.96% (95%CI=-0.57%~6.50%). The %Δsize ranged from − 29.03% to 34.78%.

**Conclusions:**

CTV expanding by 5.4 mm from radiographic GTV could include all pathologic microinvasive lesions in the radiotherapy of HCC. Liver cirrhosis was correlated with microinvasion and were independent predictive factor of microinvasion in HCC.

## Background

Primary liver cancer is one of the most prevalent tumors that seriously threaten human health [1]. China is a prominent nation of liver cancer, as it encompasses approximately 50% of the worldwide total cases [2]. Hepatocellular carcinoma (HCC) constitutes a significant majority of primary liver cancer instances, ranging from 75 to 85%. Surgical resection was previously regarded as the sole definitive approach for HCC, the insidious onset of the disease restricts the eligibility of surgical resection to a mere 20–30% of patients initially diagnosed with HCC. Furthermore, certain patients are unable to undergo invasive treatments due to underlying medical conditions. Consequently, radiotherapy serves as a non-invasive alternative for treatment.

In recent years, the remarkable advancements in radiotherapy technology and equipment have led to comparable efficacy of radiotherapy in treating small liver cancer when compared to traditional surgical interventions or radiofrequency ablation. The integration of radiotherapy with other treatment modalities has emerged as a novel therapeutic approach for advanced HCC, establishing radiotherapy as a significant treatment modality for HCC [3–9]. A crucial principle in the design of radiotherapy fields for liver cancer involves maximizing the regenerative capacity of the residual liver tissue adjacent to the tumor. Consequently, the precision of the radiotherapy target assumes paramount significance.

The gross tumor volume (GTV) in the target area of radiation therapy is usually defined in enhanced CT scan with reference to MR data. The clinical target volume (CTV) means visible lesion by radiograph or microscope, which is a highly risk area of tumor recurrence and metastasis, also known as subclinical focus. The accurate definition of CTV is a crucial aspect in achieving precision radiotherapy for liver cancer. It typically relies on the understanding of the tumor’s biological behavior and the clinical expertise of the radiotherapy physician.

Currently, there is no standardized consensus regarding the boundary of CTV for radiotherapy in HCC. Several studies have proposed different expansion ranges, such as that GTV outward expansion of 10–15 mm formed CTV in conventional radiotherapy [10–12], and GTV outward expansion of 5–7 mm formed CTV in stereotactic radiotherapy [3, 6, 7]. However, there is insufficient evidence to confirm whether these ranges effectively encompass both gross tumor lesions and subclinical lesions. The Precise Radiotherapy Study Group of the Chinese Medical Doctor Association suggests that CTV in HCC should be formed by 2–4 mm outward expansion from the GTV [13], which is based on a study by Wang et al. in 2010 who found that the distance between tumor lesions and subclinical lesions was no more than 4 mm, with an average of 1.64 mm [14]. It is believed that 4 mm expansion from GTV can include 100% of microinvasive lesions. However, this study only focused on single lesions of HCC and did not investigate the relationship between other features related to microinvasion and prognosis in HCC. Furthermore, it doesn’t take into account that the pathologic size of the tumor is not equal to the radiographic size. Therefore, the evidence supporting the expansion of CTV remains insufficient. In our study, we analyzed the correlation between various clinical features and microinvasion of HCC, and determined the distance from visible lesions of HCC to subclinical lesions, which aims to provide clinical evidence for establishing the expansion boundary from GTV to CTV in the radiotherapy target area for HCC.

## Methods

### Study design and patients

Liver cancer cases admitted to our hospital from July 2019 to November 2021 were included in this study. Inclusion criteria were: (1) Age between 18 and 80 years; (2) Clinical or pathological diagnosis of HCC, including newly diagnosed and intrahepatic recurrence patients who had not received previous treatment for the certain lesion; (3) Child-Pugh grade A or B, Performance Status (PS) score 0 to 2; (4) Adequate surgical resection range, with normal liver tissue surrounding the tumor being more than 1.0 cm; (5) Absence of serious lesions in the heart, lungs, liver, kidneys, brain, bone marrow, or other organs. Exclusion criteria were: (1) Postoperative pathologic type was not HCC; (2) The patient’s condition was not suitable for surgery.

All research was conducted in accordance with both the Declarations of Helsinki and Istanbul. The research protocol was approved by the Ethics Committee of our hospital. Informed consent was given in writing by all subjects.

### Clinical factors assessments

**Fig. 1:**
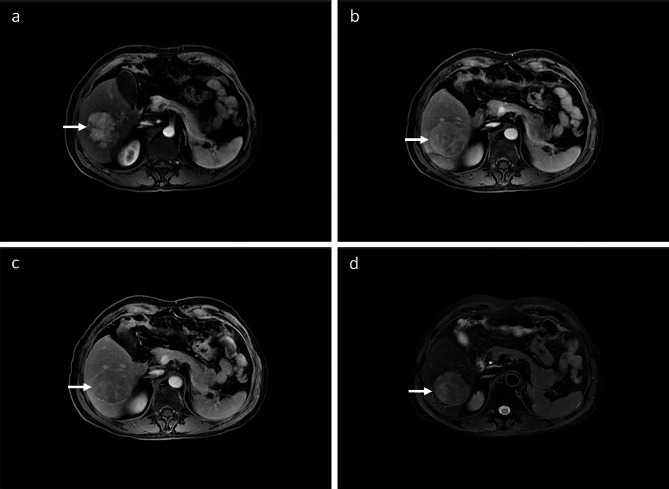
Typical imaging manifestations of HCC. (white arrows) (**a**) Arterial phase. (**b**) Portal phase. (**c**) Delayed phase. (**d**) T2WI

All patients were required to undergo a comprehensive medical history collection, physical examination, and a series of tests including routine blood tests, liver function tests, kidney function tests, coagulation function tests, as well as tests for AFP, CEA, CA19-9, HBV DNA copies, and HBV and HCV infection status within 2 weeks prior to surgery. Additionally, abdominal enhanced MRI (Discovery MR750 3.0T; GE, Waukesha, USA) with contrast media of Gd-EOB-DTPA or CT scans (Aquilion One 320; Toshiba, Tokyo, Japan) with contrast media of ioversol were performed within one month before surgery to determine the extent of intrahepatic lesion invasion. Chest CT, ECT, skull MRI, or PET-CT scans were conducted to confirm the presence of extrahepatic metastasis and determine the clinical stage of HCC. All tests and examinations were conducted at the Department of Laboratory Medicine, Department of Radiology, and Department of Nuclear Medicine of our hospital in Guangzhou, China. Clinical data of HCC patients who underwent surgical resection were collected, including gender, age, PS score, Child-Pugh score, HBV DNA copies, serum AFP value, peripheral blood platelet count, neutrophil-to-lymphocyte ratio (NLR), liver function, tumor size, tumor number, tumor location, vascular invasion, lymph node metastasis, extrahepatic metastasis, Barcelona Clinic Liver Cancer (BCLC) Stage, TNM stage, envelope condition, boundary condition, and more. The clinical diagnosis of HCC primarily relied on the “fast-in and fast-out” enhancement mode of dynamic enhanced MRI scanning [15–17], and the typical imaging manifestations of HCC were illustrated in Fig. 1. In this study, multi-parameter MRI was employed to assess whether HCC had invaded the portal vein, the hepatic vein trunk and its branches, and whether there was lymph node metastasis in the abdominal cavity or retroperitoneal space.

### Definition of pathologic factors

**Fig. 2:**
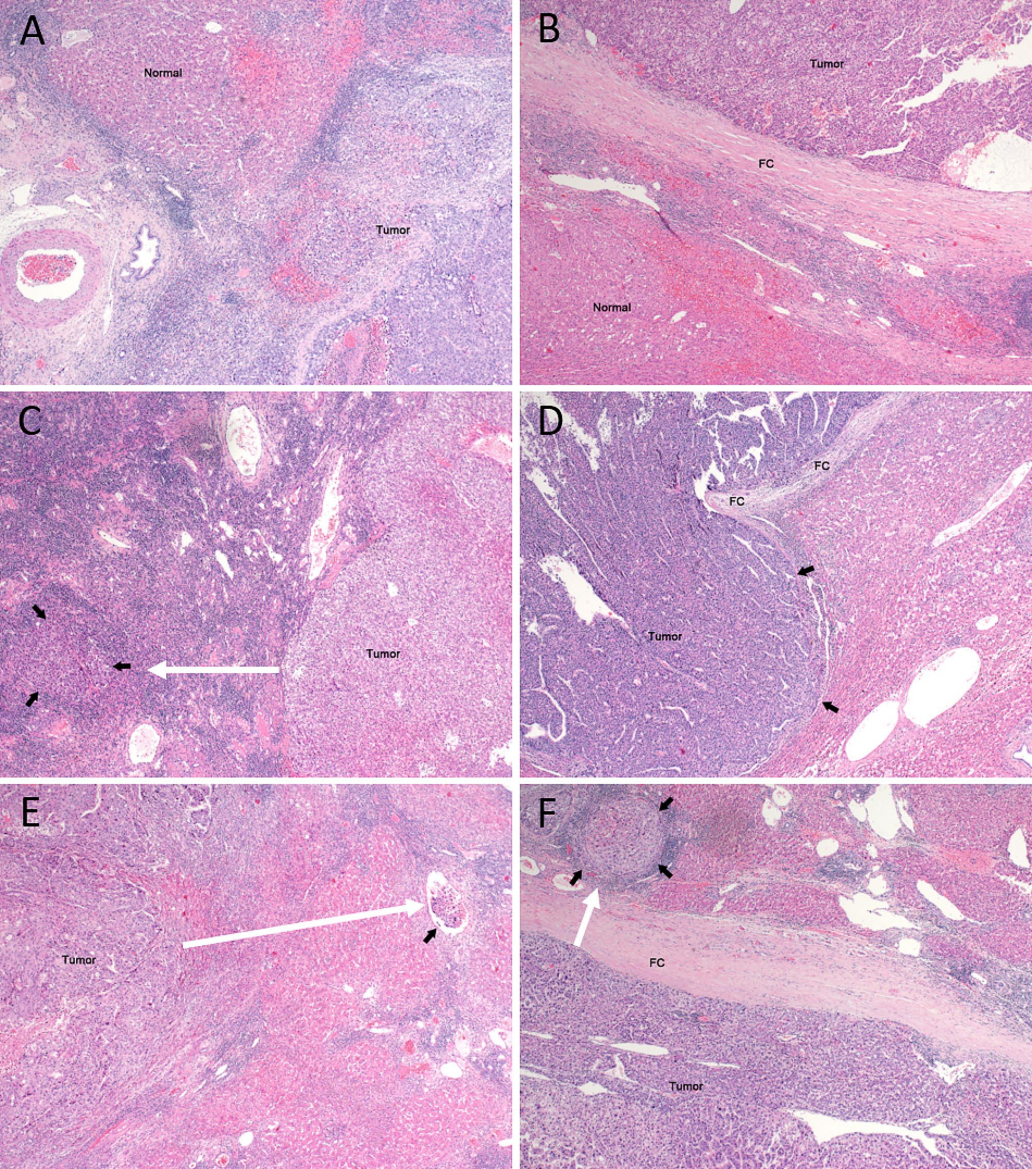
Observations of microinvasion from HCC. Microinvasion distance (white arrows). (**A**) Absent tumor capsule without microinvasion. (**B**) Presence of tumor capsule without micro-capsular invasion. (**C**) Absent tumor capsule with microinvasion (black arrows). (**D**) Presence of tumor capsule with intracapsular invasion (black arrows). (**E**) Absent tumor capsule with microvascular invasion (black arrows). (**F**) Presence of tumor capsule with extracapsular growth (black arrows). FC, fibrous capsule. (100×)

HCC specimens were promptly fixed within 30 min after resection and stored in a 10% formalin solution for 12–24 h. The surrounding area of the HCC was considered representative of the tumor’s biological behavior. Sampling was performed according to the baseline sampling method for HCC [2], with the actual sites and quantities determined based on the tumor’s diameter and number [18, 19]. All surgical specimens submitted for examination were thoroughly observed, with a focus on describing tumor size, number, color, texture, relationship with blood vessels and bile ducts, capsule status, surrounding liver tissue lesions, cirrhosis, distance from the tumor to the surgical margin, and margin status, among others. Microscopic observation and description focused on the Ki-67 value, degree of HCC differentiation, scope and degree of interstitial fibrosis, as well as the growth pattern of HCC, including peri-cancerous invasion, capsule invasion, microvascular invasion (MVI), and satellite nodules. MVI referred to the presence of cancer cell nests in the vascular lumen lined with endothelial cells under a microscope [20, 21]. The tumor microinvasion distance was observed under a microscope (Olympus BX51; Olympus, Tokyo, Japan), and the distance between the microinvasion focus and the primary tumor margin was measured. The size of the microinvasion focus was measured using a moving microscope scale. The microinvasion distance of HCC under the microscope was shown in Fig. 2. If there were multiple microinvasion foci, they were recorded based on the farthest distance of microinvasion. Microinvasion is defined as microscopic subclinical lesions that are connected to tumor or nodules separate from the main tumor in the adjacent tissues, excluding nodules which are visually visible in the MRI or CT images.

Liver cancer is frequently accompanied by varying degrees of chronic viral hepatitis or cirrhosis, and the evaluation for histological grading and staging of chronic viral hepatitis is recommended using the Scheuer score system and Chinese criteria [22–24].

### Definition of features

**Fig. 3:**
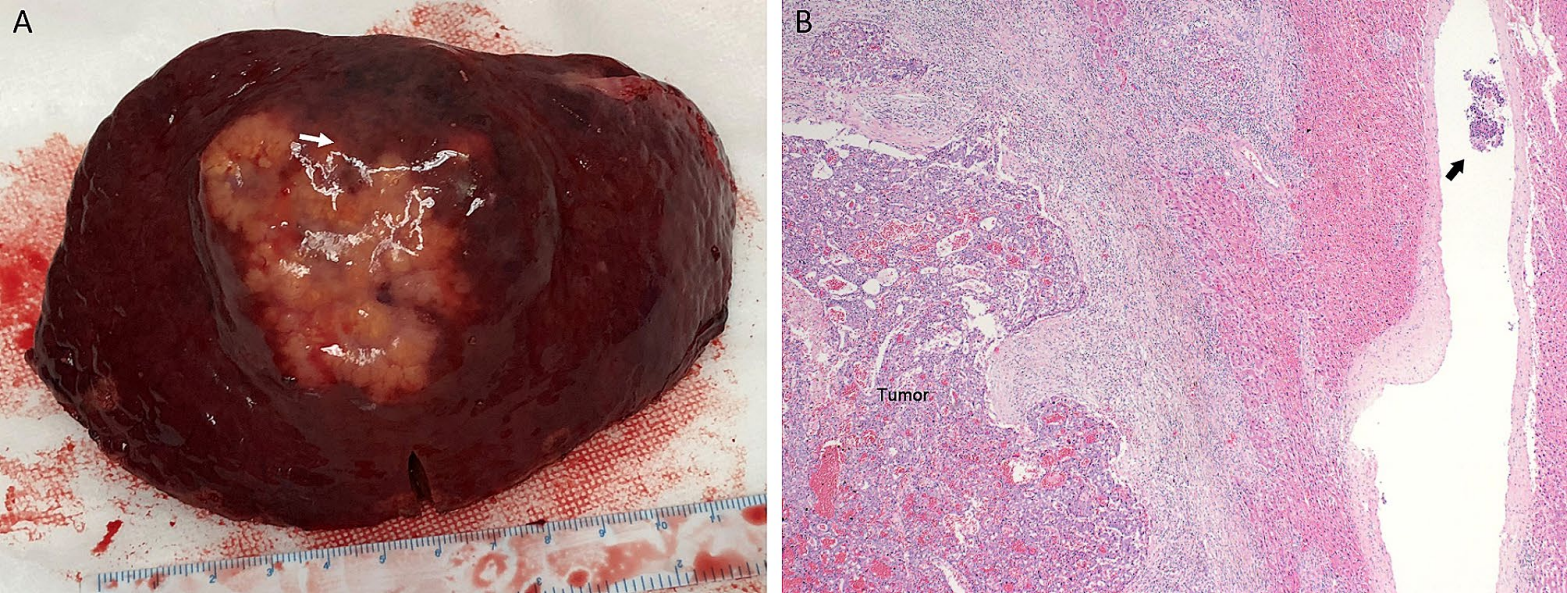
Cirrhosis and microvascular invasion of HCC. (**A**) Presence of cirrhosis and tumor capsule with intracapsular invasion. (**B**) Presence of MVI (black arrows) (100×)

The maximum image diameter was determined by the radiologist based on MRI or CT scans, while the maximum pathologic diameter was determined by the pathologist on the excised specimen. The tumor boundary was determined according to the surgical records of the surgeon. The border area of the tumor was observed visually. If there was visible fibrous tissue encapsulating the tumor or a clear and regular boundary between the cancerous focus and the adjacent non-cancerous tissue, it was defined as ‘border clear’. If there was no obvious capsule or the boundary was unclear, it was defined as ‘border ill-defined’. The integrity of the capsule was recorded by the pathologist under the microscope. Vascular invasion and cancer embolus were defined based on MR or CT findings. MVI was observed by the pathologist. Figure 3 shows a case of MVI.

### Correlation between radiographic and pathologic size

The radiographic tumor size (RTS) was determined as the largest diameter observed in MRI or CT scans. On the other hand, the pathologic tumor size (PTS) was measured as the largest diameter of the tumor mass before formalin fixation of the gross surgical specimen, as recorded in the pathologic report. In cases where patients had multiple hepatic tumors, only the largest tumor was considered for analysis. Tumor dimensions were assessed through direct visual inspection and measurement using a metric ruler with millimeter demarcations. To compare the RTS and PTS, a percent of size difference (%Δsize) was calculated for each patient using the formula:

%∆size = (radiographic size - pathologic size) / radiographic size.

This allowed for the evaluation of the discrepancy between the two measurements. A rank correlation analysis was conducted to examine the linear relationship between pathologic and radiographic size.

To ensure consistency and minimize bias between different viewers, all slides and images were examined by the same two experienced pathologist or the same two experienced radiologist individually for each routine section.

### Statistics

Data analysis was performed using SPSS 25.0 software. Quantitative data with a normal distribution were presented as Mean ± SD, and a T-test or F-test was used to compare groups. Categorical data were presented as the number of cases and percentages. The comparison between disordered groups was conducted using the chi-square test or Fisher’s exact probability test, while the comparison between ordered groups was analyzed using the chi-square test or rank sum test.

To analyze the correlation between different features and the microinvasion distance of HCC, multivariate logistic regression analysis was performed. The goodness of fit of the regression model was assessed using the Hosmer-Lemeshow (H-L) goodness of fit test. A significance level of *P* < 0.05 was considered statistically significant.

## Results

### Microinvasion distance distribution

A total of 44 patients were enrolled, with a total of 173 liver cancer tumors and adjacent tissue specimens included in this study. Among the participants, 40 were males (90.91%) and 4 were females (9.09%). The mean age of the patients was 55.0 ± 11.5 years, ranging from 30 to 77 years. Among the patients, 40 (90.91%) had a history of chronic HBV infection, while none had a history of chronic HCV infection. Table 1 presented the microinvasion distances observed in patients with HCC. Among the total number of patients, 17 (38.63%) had varying degrees of microinvasion distance, while 27 patients (61.36%) did not show any microinvasion distance. The maximum observed microinvasion distance was 4.0 mm, with an average distance of 0.6 mm (95%CI 0.3 ~ 0.9 mm). In 95% of cases, the microinvasion distance was less than 3.0 mm. Based on these findings, it can be concluded that expanding the CTV by 4.0 mm beyond GTV should be sufficient to encompass 100% of the pathologic microinvasion lesions in HCC.

**Table 1:** Microinvasion distance distribution for HCC

Microinvasion distance (mm)	*n*	Cumulative *n*	%	Cumulative %
0.0	27	27	61.36%	61.36%
0.1–0.9	4	31	9.01%	70.45%
1.0-1.9	7	38	15.91%	86.36%
2.0-2.9	4	42	9.09%	95.45%
3.0-3.9	1	43	2.27%	97.73%
4.0-4.9	1	44	2.27%	100.00%
≥ 5.0	0	44	0.00%	100.00%

### Features association with microinvasion

The chi-square test was employed to examine the association between various categorical variables and the presence or absence of microinvasion. The results indicated that liver cirrhosis was significantly associated with microinvasion. Patients with cirrhosis had a lower risk of microinvasion compared to those without cirrhosis (47.1% vs. 77.8%, *P* = 0.036) (Table 2). Additionally, serum albumin level was found to be associated with microinvasion (*P* = 0.049). The T-test was used to assess the relationship between quantitative data and microinvasion. The analysis revealed a weak correlation between Ki-67 and microinvasion (*P* = 0.054), as presented in Table 2. However, tumor size, location, number, extrahepatic metastasis, tumor stage, MVI, histological grade, liver function status, Child-Pugh score, HBV infection status, HBV-DNA copies, AFP, CEA, CA19-9, and other features were not found to be correlated with the occurrence of microinvasion.

**Table 2:** Features association with microinvasion of HCC

	Microinvasion		χ^2^/t value	*P*
	Absent (*n* = 27)	Present (*n* = 17)		
Gender			1.06	0.304
Male	26 (96.3%)	14 (82.4%)		
Female	1 (3.7%)	3 (17.6%)		
Age (year)	57.07 ± 10.23	51.82 ± 12.99	1.49	0.143 ^*^
Ki-67 (%)	28.19%±20.55%	42.18%±26.03%	-1.98	0.054 ^*^
HBsAg (IU/mL)			0.82	0.799
<0.05	3 (11.1%)	2 (11.8%)		
0.05–250	6 (22.2%)	2 (11.8%)		
>250	18 (66.7%)	13 (76.5%)		
HBV DNA (IU/mL)			0.17	0.680
<100	11 (40.7%)	8 (47.1%)		
≥ 100	16 (59.3%)	9(52.9%)		
AFP (ug/L)			1.37	0.242
≤ 400	19 (70.4%)	9(52.9%)		
>400	8 (29.6%)	8 (47.1%)		
CEA (ug/L)			0.00	1.000
≤ 5	26 (96.3%)	16 (94.1%)		
>5	1 (3.7%)	1 (5.9%)		
CA19-9 (U/mL)			2.71	0.100
≤ 35	27 (100.0%)	14 (82.4%)		
>35	0 (0.0%)	3 (17.6%)		
PLT (10^9^/L)			0.00	1.000
<100	2 (7.4%)	2 (11.8%)		
≥ 100	25 (92.6%)	15 (88.2%)		
NLR			0.36	0.548
≤ 2	12 (44.4%)	6(35.3%)		
>2	15 (55.6%)	11 (64.7%)		
TBIL (umol/L)			0.06	0.813
≤ 23.9	27 (100.0%)	16 (94.1%)		
>23.9	0 (0.0%)	1 (5.9%)		
ALB (g/L)			3.88	0.049
≤ 35	1 (3.7%)	5(29.4%)		
>35	26 (96.3%)	12 (70.6%)		
ALT (U/L)			0.44	0.507
≤ 35	20 (74.1%)	11 (64.7%)		
>35	7 (25.9%)	6(35.3%)		
AST (U/L)			2.59	0.108
≤ 40	23 (85.2%)	10 (58.8%)		
>40	4 (14.8%)	7 (41.2%)		
PT (s)			0.11	0.743
≤ 14.5	23 (85.2%)	13 (76.5%)		
>14.5	4 (14.8%)	4 (23.5%)		
Child-Pugh Score			4.35	0.084
5	22 (81.5%)	9(52.9%)		
6	4 (14.8%)	7 (41.2%)		
7	1 (3.7%)	1 (5.9%)		
Tumor diameter (cm)			1.72	0.190
≤ 5	15 (55.6%)	6(35.3%)		
>5	12 (44.4%)	11 (64.7%)		
Number of tumors			0.48	0.488
1	18 (66.7%)	13 (76.5%)		
>1	9 (33.3%)	4 (23.5%)		
Location of tumor			2.48	0.212
Left lobe	11 (40.7%)	9(52.9%)		
Right lobe	16 (59.3%)	7 (41.2%)		
Caudate lobe	0 (0.0%)	1 (5.9%)		
Capsular			0.62	0.432
Absent	23 (85.2%)	12 (70.6%)		
Present	4 (14.8%)	5(29.4%)		
Border of tumor			0.36	0.548
Clear	21 (77.8%)	11 (64.7%)		
Ill-defined	6 (22.2%)	6(35.3%)		
Vascular invasion			0.63	0.429
Absent	16 (59.3%)	8 (47.1%)		
Present	11 (40.7%)	9(52.9%)		
Lymph node metastasis			1.06	0.304
Absent	26 (96.3%)	14 (82.4%)		
Present	1 (3.7%)	3 (17.6%)		
Extrahepatic metastasis			0.00	1.000
Absent	25 (92.6%)	16 (94.1%)		
Present	2 (7.4%)	1 (5.9%)		
Ascites			0.00	1.000
Absent	22 (81.5%)	14 (82.4%)		
Present	5 (18.5%)	3 (17.6%)		
BCLC stage			0.77	0.737
A	13 (48.1%)	7 (41.2%)		
B	3 (11.1%)	1 (5.9%)		
C	11 (40.7%)	9(52.9%)		
Cirrhosis			4.38	0.036
Absent	6 (22.2%)	9(52.9%)		
Present	21 (77.8%)	8 (47.1%)		
MVI			3.66	0.165
M0	16 (59.3%)	7 (41.2%)		
M1	9 (33.3%)	5(29.4%)		
M2	2 (7.4%)	5(29.4%)		
Pathologic grade of tumor			1.00	0.838
Well differentiation	1 (3.7%)	0 (0.0%)		
Median differentiation	19 (70.4%)	11 (64.7%)		
Poor differentiation	7 (25.9%)	6 (35.3%)		

*, analyzed by T-test; other data was analyzed by chi-square test

Abbreviations: AFP, alpha fetal protein; CEA, carcinoembryonic antigen; CA19-9, carbohydrate antigen 19-9; PLT, blood platelet; NLR, neutrophil-to-lymphocyte ratio; TBIL, serum total bilirubin level; ALB, serum albumin level; ALT, serum alanine aminotransferase level; AST, serum aspartate aminotransferase level; GGT, serum γ-glutamyl transferase level; ALP, serum alkaline phosphatase level; PT, prothrombin time; MVI, microvascular invasion; BCLC, Barcelona clinical liver cancer

### Predictors to microinvasion of HCC

Independent variables with a significance level of *P* < 0.10, obtained through simple correlation analysis methods such as T-test, rank sum test, and chi-square test, were included in a multivariate logistic regression analysis model. These variables included liver cirrhosis (*P* = 0.036), ALB (*P* = 0.049), Ki-67 (*P* = 0.054), and Child-Pugh score (*P* = 0.084). The multivariate logistic regression analysis revealed that liver cirrhosis was significantly associated with microinvasion (H-L goodness of fit, χ^2^ = 8.02, *P* = 0.432), as presented in Table 3. HCC patients with liver cirrhosis had a significantly lower risk of microinvasion (*OR* = 0.09, 95%CI = 0.02 ~ 0.50, *P* = 0.006). Patients with liver cirrhosis had an average microinvasion distance of 0.5 mm (95%CI 0.1 ~ 0.8 mm). Patients without liver cirrhosis had an average microinvasion distance of 0.8 mm (95%CI 0.2 ~ 1.3 mm). Based on these findings, it can be concluded that expanding the CTV by 0.8 mm beyond GTV should be sufficient to encompass 95% of the pathologic microinvasion lesions in HCC with cirrhosis, while HCC patients without cirrhosis need to expand by 1.3 mm.

**Table 3:** Variables associated with microinvasion following multivariate analysis in HCC

	Microinvasion	
	OR(95% CI)	*P*
Cirrhosis	0.09(0.02 ~ 0.50)	0.006
ALB	0.03(0.001 ~ 1.43)	0.075
Ki-67	37.71 (0.77 ~ 1853.34)	0.068
Child-Pugh score	0.62(0.08 ~ 4.52)	0.634

### Tumor size between radiographic and pathologic samples

**Fig. 4:**
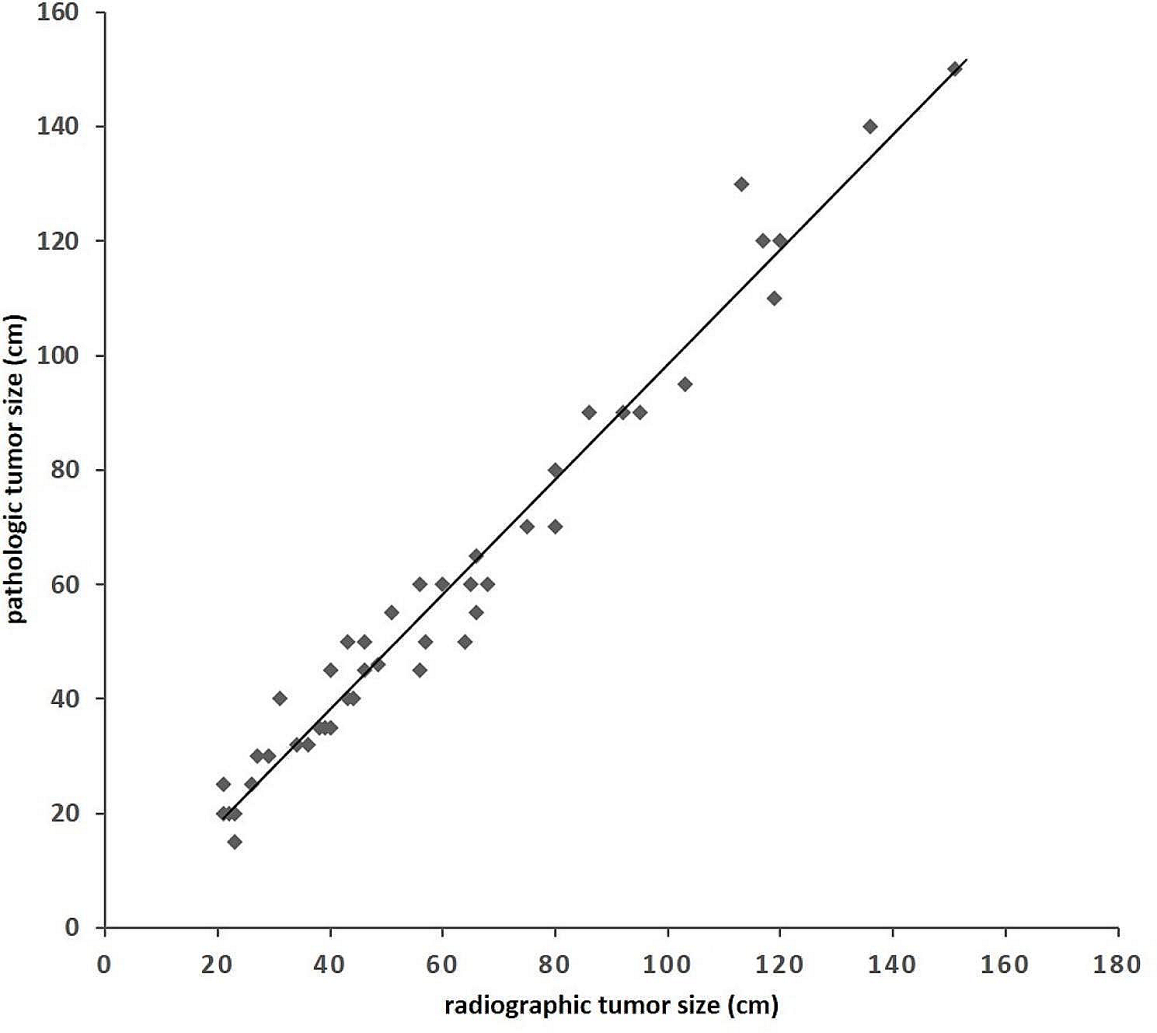
Correlation between radiographic and pathologic tumor size for HCC. The line of unity is shown for reference

Figure 4 demonstrated a strong correlation between RTS and PTS (*r* = 0.979; *P*<0.001). The radiographic size was larger than that of the pathologic sample in 63.6% (28/44) of tumors, smaller in 29.6% (13/44) of tumors, and equal in 6.8% (3/44) of tumors. Overall, the pathologic sample size was slightly smaller than the radiographic size. However, there were no significant differences between the two groups (*P* = 0.823), as indicated in Table 4.

**Table 4:** Radiographic and pathologic tumor size of HCC

	*n*	RTS (mm)	PTS (mm)	Difference and 95%CI (mm)	t value	*P*	%∆Size	
							Mean ± SD	95%CI
All	44	61.28 ± 33.70	59.66 ± 34.33	1.63(-12.79 ~ 16.04)	0.22	0.823	2.96 ± 11.60	-0.57 ~ 6.50
Scaning								
MR	39	58.11 ± 32.46	56.79 ± 33.10	1.32(-13.47 ~ 16.11)	0.18	0.859	2.12 ± 11.14	-1.49 ~ 5.74
CT	5	86.00 ± 36.53	82.00 ± 39.47	4.00(-51.45 ~ 59.45)	0.17	0.872	9.48 ± 14.66	-8.71 ~ 27.68
RTS (mm)								
<50	21	34.31 ± 9.34	33.81 ± 10.39	0.50(-5.66 ~ 6.66)	0.16	0.871	1.73 ± 14.00	-4.64 ~ 8.11
50 ~ 100	16	69.81 ± 13.44	65.63 ± 14.82	4.19(-6.03 ~ 14.40)	0.84	0.409	6.15 ± 9.15	1.28 ~ 11.02
>100	7	122.71 ± 15.88	123.57 ± 18.42	-0.86(-20.87 ~ 19.17	-0.09	0.927	-0.65 ± 7.70	-7.78 ~ 6.48

Abbreviations: RTS, radiographic tumor size; PTS, pathologic tumor size

The average radiographic size was 61.3 mm (range 21.0–151.0 mm), while the average pathologic size was 59.7 mm (range 15.0–150.0 mm), as shown in Table 4. Tumors were overestimated by average 1.6 mm (95% CI = − 12.8 ~ 16.0 mm) on radiographic compared to pathologic size. The mean percentage difference in size (%Δsize) was 2.96% (95% CI = -0.57%~6.50%), with a range of -29.03%–34.78%. To ensure adequate coverage of gross lesions, a margin of ‘pathologic microinvasive distance × 106.50%’ would have covered 95% of the radiographic lesions, while a margin of ‘pathologic microinvasive distance × 134.78%’ would have covered 100% of the radiographic lesions. As previously mentioned, we found that the pathologic microinvasion distance was less than or equal to 4.0 mm in 100% of cases. Therefore, using a 4.3 mm margin around the radiographic tumor for radiation therapy would have covered 95% of the subclinical focus, and a 5.4 mm margin would have covered 100% of the subclinical focus.

In our study, we found that factors such as MRI or CT scanning and radiographic size had no significant impact on the differences between radiographic and pathologic tumor size. More details can be found in Table 4.

## Discussion

The accurate definition of the target area is crucial for the successful implementation of precision radiotherapy for liver cancer. Conventional imaging can be used to determine the target area for tumor radiotherapy, and the dose distribution can be tailored to match the shape of the tumor using external irradiation beams. However, determining a reasonable and precise target area requires comprehensive consideration of all relevant uncertainties and clinical practices. This study aimed to analyze the relationship between various features of HCC and microinvasion, and to clarify the distance between visible tumor lesions and subclinical lesions. The findings provide a clinical basis for expanding the boundary of the GTV to the CTV in liver cancer radiotherapy. This approach helps minimize the radiation dose to the surrounding liver tissue while achieving precise dose carving and radiotherapy.

However, there is currently no standardized expansion boundary from GTV to CTV in the radiotherapy target area for HCC. MH Wang et al. [14] reported that the microinvasion distance in 96.1% of HCC patients was ≤ 2.0 mm, and the distance from the lesion to the subclinical lesion did not exceed 4 mm, with an average of 1.64 mm. They suggested that the outward expansion of 4 mm from GTV to CTV could include 100% microinvasive lesions. WH Wang et al. [25] found that the microinvasion distance was related to the histological grade of HCC. The mean microinvasion distance was (0.0 ± 0.1) mm (range 0 ~ 0.2 mm) in grade 1 patients, (0.9 ± 0.9) mm (range 0 ~ 4.5 mm) in grade 2 patients, and (1.9 ± 1.9) mm (range 0 ~ 8.0 mm) in grade 3 patients. They concluded that for grade 1, 2, and 3 patients, expanding from the GTV to the CTV by 0.2 mm, 4.5 mm, and 8.0 mm, respectively, could include all microinvasive lesions. In our study, the average microinvasion distance was 0.6 mm, and the pathologic microinvasion distance in 95% of cases was less than 3.0 mm. The farthest microinvasion distance observed was 4.0 mm. CTV expanded by 4.0 mm from GTV could include 100% of pathologic microinvasion lesions.

However, the prognosis of liver cancer is influenced by various factors, such as tumor characteristics, patient’s general condition, and liver function. The studies conducted by Wang et al. and Wang et al. only focused on certain features associated with the prognosis of HCC. MH Wang et al. found that factors like PLT, AFP level, maximum tumor diameter, portal vein cancer thrombus, and TNM stage were related to the microinvasion distance of the tumor. On the other hand, gender, tumor envelope status, cirrhosis, and Edmondson-Steiner grade did not show any statistical significance in relation to tumor microinvasion. WH Wang et al. reported that the microinvasion distance was associated with the histological grade of the tumor, but not with age, sex, hepatitis status, AFP level, tumor size, tumor stage, PLT, and liver function markers such as AST, ALT, GGT, ALB, BIL, and PT. These studies did not investigate the correlation between HBV-DNA copies, Child-Pugh score, NLR, tumor number, tumor location, tumor boundary, lymph node metastasis, extrahepatic metastasis, ascites, MVI, Ki-67, and other important biological and clinical features related to prognosis and microinvasion. Therefore, the evidence supporting the expansion of CTV is insufficient. Our study included a wider range of biological and clinical features, including patient characteristics, liver function, and tumor types. Multivariate logistic regression analysis revealed that liver cirrhosis was correlated with tumor microinvasion distance. However, no correlation was found between microinvasion distance and PLT, AFP level, maximum tumor diameter, portal vein thrombus, tumor stage, or histological grade.

There was no study revealed the effect of liver cirrhosis on pathological microinvasion distance of liver cancer. In previous studies, researchers tended to believe that liver cirrhosis is commonly associated with HCC and has been shown to have a negative impact on long-term prognosis. HCC patients without cirrhosis generally have a better prognosis compared to those with cirrhosis [26]. Prognostic factors for OS in HCC patients with cirrhosis include tumor diameter, BCLC stage, hepatitis B surface antigen (HBsAg), positive HCV antibodies, elevated creatinine, and elevated total bilirubin. Most HCC patients in China have some degree of liver cirrhosis, and the effect of cirrhosis on the long-term prognosis after hepatectomy is still unclear. Wu Z et al. [27] found that the RFS and OS rates were significantly lower in the cirrhosis group compared to the non-cirrhosis group. Subgroup analysis showed that among patients with BCLC stage 0-B disease, RFS and OS were significantly lower in those with cirrhosis. While in patients with BCLC stage C disease, there was no significant difference between those with and without cirrhosis. The independent risk factors for RFS and OS differ between patients with and without cirrhosis. The mechanism of OS effected by cirrhosis has not been well explained. The cause of death in liver cancer with cirrhosis may be mainly related to poor liver function, rather than tumor. Chen et al. [28] reported that HCC patients with cirrhosis have smaller tumor diameter, while those without cirrhosis have larger tumors. We found that HCC patients with cirrhosis showed a shorter microinvasion distance than those without cirrhosis. There was no difference between patients with and without cirrhosis grouped by AFP, PLT, TBIL, ALB, Ki-67, Child-Pugh score, tumor diameter, BCLC stage, and other variables. Currently, the relationship between pathologic microinvasion and cirrhosis is not fully understood. When designing a radiotherapy plan, CTV could be expanded beyond the GTV by a little smaller margin in HCC patients with cirrhosis.

HCC is a type of hyper vascular neoplasm. After surgical resection, the tumors experience a loss of blood supply, resulting in tumor shrinkage [29]. This means that the size of the tumor seen on radiographic images cannot be directly translated to the size of the tumor in pathologic specimens. Several studies have been conducted to compare radiographic size with pathologic size in HCC. Chen et al. [28] found that the radiographic size was larger than or equal to the pathologic size in 110/174 (63.2%) tumors, and smaller in 64/174 (36.8%) tumors. The median difference in size (%Δsize) was 3.3%. When planning radiation therapy, utilizing a 15 mm margin around the radiographic tumor would have covered 90% of the pathologic gross lesions, while a margin of 21 mm would have covered 95% of the lesions. For tumors smaller than 50 mm, utilizing a 3 mm margin would have covered 90% of the lesions, a 5 mm margin would have covered 95%, and a 15 mm margin would have covered 100%. They found that radiographic size of 3–5 cm and pathologic Grade I with clear boundaries, overestimation of tumor size by CT in univariate analysis. But multivariate analysis shown none of the variables such as radiographic tumor size, pathologic grading, tumor boundary or cirrhosis were found to be predictive of larger discrepancy. In our study, univariate analysis did not find the effect of CT or MR Scanning, radiographic tumor size, cirrhosis, and other variables, on pathological tumor size. Similar results were reported by Kelsey et al. [30], which radiographic tumor size was equal to or larger than pathologic size in 22 out of 27 cases (81%), and smaller in 5 cases (19%), with a mean %Δsize of 3.39%. Radiation therapy utilizing a 5 mm margin around the radiographic tumor would have covered 93% of the pathologic gross lesions, while a 10 mm margin would have covered 100%. According to our study, we found that the radiographic size of HCC on tri-phase hepatic MRI or CT correlated well with the pathologic size. Utilizing the radiographic tumor for radiation planning would have covered 70.45% (31/44) of the pathologic gross lesions. Tumors were overestimated by 1.6 mm on radiographic compared to pathologic size, with the %Δsize ranged from − 29.03–34.78%. Using a margin of ‘pathologic microinvasive distance × 134.78%’ would have covered 100% of the radiographic lesions. Additionally, we found that the pathologic microinvasion distance was less than or equal to 4.0 mm in 100% of cases. Therefore, using a 5.4 mm margin around the radiographic tumor for radiation therapy would have covered 100% of the subclinical focus.

## Conclusions

The findings of this study indicated that when planning radiotherapy for HCC, a CTV expansion of 5.4 mm from the radiographic GTV should be considered to encompass all pathologic microinvasive lesions. Liver cirrhosis was found to be associated with shorter microinvasion distance and were identified as independent predictor of microinvasion in HCC.

## Data Availability

No datasets were generated or analysed during the current study.

## Abbreviations

HCC: hepatocellular carcinoma
CTV: clinical target volume
GTV: gross tumor volume
PS: Performance Status
BCLC: Barcelona Clinic Liver Cancer
MVI: microvascular invasion
AFP: alpha fetal protein
CEA: carcinoembryonic antigen
CA19-9: carbohydrate antigen 19 − 9
PLT: blood platelet
NLR: neutrophil-to-lymphocyte ratio
TBIL: serum total bilirubin level
ALB: serum albumin level
ALT: serum alanine aminotransferase level
AST: serum aspartate aminotransferase level
GGT: serum γ-glutamyltransferase level
ALP: serum alkaline phosphatase level; PT: prothrombin time
MVI: microvascular invasion
RTS: radiographic tumor size
PTS: pathologic tumor size
OS: overall survival
RFS: recurrence-free survival
